# Effect of Contextual Interference and Differential Learning on Motor Skill Development and Motivation in Novice Basketball Players

**DOI:** 10.1002/ejsc.70061

**Published:** 2025-09-30

**Authors:** Ghazal Shamshiri, Davoud Fazeli, GholamHossein Nazemzadegan

**Affiliations:** ^1^ Department of Sport Sciences Faculty of Education and Psychology Shiraz University Shiraz Iran

**Keywords:** dynamic systems, motor learning, stochastic resonance, variability

## Abstract

This study aimed to investigate the effect of three practice orders in the contextual interference and differential learning frameworks on motivation and motor learning of novice basketball players. For this purpose, 84 female students were divided into seven groups (progressive practice–contextual interference framework, random practice–contextual interference framework, blocked practice–contextual interference framework, progressive variations‐differential learning, stochastic variation‐differential learning, predictable variation‐differential learning, and control). After a pretest (18 trials), participants practiced the basketball jump shot for three consecutive days according to their grouping (3 blocks of 18 attempts each day). The contextual interference groups varied in shooting distances: random (different distance each trial), blocked (same distance all day), or progressive order (blocked first day, serial second day, random third day). The differential groups varied in body positions: random (unpredictable changes), blocked (predictable daily changes from head to foot), or progressive order (blocked first day, serial second day, random third day). After the last block of the third day of practice, participants completed the motivation questionnaire, as motivation can affect motor learning. Three days after the acquisition, participants performed two retention tests with fixed and variable targets. Additionally, a transfer test including 18 trials from a different distance was performed. Results indicated that the blocked practice–contextual interference group performed more accurately than other groups during acquisition. However, a higher transfer was observed for differential learning groups. Additionally, differential groups and random practice–contextual interference group showed higher motivation. These findings were discussed according to the role of noise in the exploration of the solution space of the task.

## Highlights

- Practice methods compared: This study examined contextual interference (blocked, random, progressive) and differential learning (predictable, stochastic, progressive) in novice basketball players to assess effects on skill acquisition and motivation.
- Acquisition versus transfer: Blocked practice led to higher accuracy during acquisition, but differential learning groups outperformed others in transfer, showing stronger adaptability.
- Motivation outcomes: Random practice (CI) and all differential learning groups reported higher intrinsic motivation (interest, enjoyment, effort) compared to blocked and progressive CI groups.
- Key implication: Differential learning, by introducing optimal variability (“noise”), may better enhance both long‐term skill transfer and learner motivation than contextual interference.

## 1 Introduction

Because practice and experience are foundations of motor learning, enhancing the practice environment can potentially lead to improvements in motor learning. Consequently, researchers have been exploring various methods to enhance learning by enriching the training environment. Two key approaches in this area are differential learning, which emphasizes high variability and nonrepetitive movement patterns to promote self‐organization and adaptability (W. I. Schöllhorn 2016), and contextual interference, which involves practicing multiple tasks (or different versions of the same task) in a random order to enhance long‐term retention and transfer of skills despite initial performance decrements (Brady 2008). Both methods are based on the core idea of movement variability, which is a critical concept in motor learning and control and has been explored from various angles (W. I. Schöllhorn 2016).

According to traditional cognitive views, variability in movement was considered noise and deemed detrimental to performance and learning. In contrast, the dynamic systems view regards variability in movement as a beneficial factor that enhances movement flexibility (Davids et al. 2006). According to this view, introducing variability in movement patterns through the induction of noise can facilitate learning. This perspective is known as differential learning (W. I. Schöllhorn et al. 2006, 2004). In addition, it is believed that the parameters enhanced by variability depend on the level at which it is introduced (at the target level or the motor execution level) (Ranganathan and Newell 2010, 2013). These contrasting viewpoints have led to the development of different methods when considering variability in practice. The traditional views suggest minimizing variability in movement patterns to enhance performance consistency, but they propose introducing variability at the target level to improve generalizability (Ranganathan and Newell 2013). This concept gives rise to the variability of practice hypothesis (R. A. Schmidt 1975) and contextual interference (Shea and Morgan 1979).

The contextual interference effect is a phenomenon wherein the interference generated through performing multiple skills or different variations of a skill during the acquisition phase can reduce overall practice performance but enhance participants' performance during retention and transfer tests. The degree of interference depends on how tasks are organized or presented within a practice schedule (Shea and Morgan 1979). In cases where a participant practices only one skill or practices skills in a blocked order (referred to as blocked practice), the interference is minimal. Conversely, when practicing several different yet related skills in a single session (known as random order), the interference is heightened. Additional practice schedules include serial practice, characterized by a fixed and repeating sequence of task variations that produces a moderate level of interference, and the progressive practice schedule, in which the degree of interference is systematically increased over successive sessions (progressing from blocked to serial to random practice), as outlined by Porter and Magill (2010). Although numerous studies have shown the contextual interference effect, and their findings have been synthesized in several review articles (Brady 2008; Magill and Hall 1990), it is important to note that much of the research evidence supporting the contextual interference effect comes from studies employing laboratory tasks (Buszard et al. 2017; Farrow and Buszard 2017). In contrast, when examining the contextual interference effect on field sport tasks, conflicting results have emerged, leading to less consensus in their findings (Buszard et al. 2017; Farrow and Buszard 2017). Consequently, further research is necessary to explore contextual interference using real sports tasks to gain a more comprehensive understanding.

In contrast to the concept of practice variability and contextual interference, there is an alternative practice method known as differential learning, which emerged from the dynamic systems perspective (W. I. Schöllhorn et al. 2006). This approach, developed under the guidance of dynamic systems theory, challenges the notion of repeating movements based on an ideal movement pattern. Differential learning emphasizes the importance of variability in the learning process and involves incorporating noisy changes in the movement pattern. For example, changing the initial body positions (putting with one hand, closing one eye during putting, and freezing a joint or a body part) during the putting task can be considered differential learning, and changing the distance from the hole (in a random or blocked order) may be considered contextual interference (Mousavi et al. 2024). Consequently, during the skill acquisition phase, the differential practice involves executing movements without repetitions, utilizing noisy changes to enhance the process of finding the optimal solution for each learner. In contrast to the contextual interference, which focuses on performing movements according to a predefined optimal solution, differential learning recognizes the impossibility of performing two identical movements in real settings. Therefore, the process of identifying a common and ideal movement pattern for all learners becomes futile within this framework (W. I. Schöllhorn et al. 2006). Instead, differential learning utilizes increased fluctuations as an active tool to guide the system toward instability, where less energy is required to attain a new stable state (Savelsbergh et al. 2010). Additionally, from a differential learning point of view, there is a nonlinear inverted U‐shaped relationship between the amount of variation during acquisition and retention/transfer (Beckmann et al. 2010; W. I. Schöllhorn et al. 2009). Low and high levels of noise during acquisition may result in nonoptimal learning. According to this theory, the optimal level of variation would be close to the noise level associated with differential learning (W. I. Schöllhorn et al. 2009). From this perspective, contextual interference could be considered a nonoptimal level of variation compared with differential learning.

Despite the theoretical appeal of differential learning (DL), several authors have raised substantive critiques of its foundational claims. Künzell and Hossner (2012, 2013), Hossner et al. (2016a, 2016b), and Tassignon et al. (2021) have argued that DL's core recommendations—such as minimizing repetition and withholding feedback—are not consistently supported by empirical data and that “noise” in movement lacks a unified operational definition. These critiques point to methodological limitations (e.g., small sample sizes, lack of rigorous control groups) and question whether observed performance gains truly reflect stochastic resonance or simply the effects of novelty and heightened attention. We, therefore, adopt DL as a promising but still contested approach within a broader spectrum of variability‐based practice methodologies.

Although contextual interference and differential learning differ both theoretically and practically, relatively few studies have directly compared these two methods. Additionally, the results of these studies are partially contradictory. Furthermore, existing research has primarily focused on investigating the short‐term effects of these methods, and it remains unclear which of these two leads to more robust learning. For instance, in a study, the immediate post‐task effects of differential learning and contextual interference on electroencephalographic brain activation patterns were examined (Henz et al. 2018). The results revealed that differential learning stimulates the somatosensory and motor systems, as well as broader areas of the cerebral cortex, in comparison to repetitive training. Additionally, it appeared that contextual interference activates specific executive‐controlled processes in the frontal cortex. Additionally, the short‐term effects of contextual interference and differential learning in a goalkeeping task were compared (Serrien et al. 2020). Results showed no significant difference between these two training methods during the retention test. These two methods were also compared in learning a golf putting task (M. Schmidt et al. 2021). In this study, a random group was compared with different amounts of variation using a differential learning method. Results showed improvement from pretest to post‐test for all groups; however, no significant difference was observed between experimental groups. In contrast, it was shown that in learning three volleyball tasks, differential learning would result in a greater learning effect than contextual interference (Apidogo et al. 2022).

Research indicates that variability in practice—whether from contextual interference or differential learning—is a critical factor in learning motor skills. According to the challenge point framework (Guadagnoli and Lee 2004), the amount of variability in a practice should match the learner's skill level. If the practice order's challenge level is not proportional to the learner's skill level, learning may be hindered. Similarly, the differential learning perspective suggests that excessive noise in practice, when the learner's internal noise level is high, can be detrimental to the learning process (W. Schöllhorn 2005; W. I. Schöllhorn et al. 2009). Both perspectives agree that variability should be proportional to skill level. Studies on contextual interference have attempted to manipulate practice variability by introducing progressive practice schedules (Beik and Fazeli 2021; Beik et al. 2022; Porter and Magill 2010). These studies found that progressive practice order—starting with blocked practice, progressing to serial practice, and culminating in random practice—leads to greater learning than traditional methods (pure random or pure blocked practice). To our knowledge, there has been no research addressing progressive differential learning or comparing this practice method with the progressive schedule using the contextual interference framework.

It has also been argued that using variability in practice can affect participants' intrinsic motivation (Beik and Fazeli 2021). For example, research showed that using a practice schedule proportional to the participants' skill level would result in higher motivation than classical random or blocked practice schedules (Beik and Fazeli 2021). These results were replicated in another study using electroencephalography (Beik et al. 2022). It is believed that variability hinders participants from getting bored by changing the task from trial to trial (Beik and Fazeli 2021). Additionally, variability may make the task more challenging for the participant and accordingly increase her/his internal motivation (Beik et al. 2022). This finding aligns with a recent theory proposed in the field of motor learning. According to the OPTIMAL learning theory (optimizing performance through intrinsic motivation and attention for learning), attention and motivation processes enhance performance and learning by strengthening the coupling of goals to actions (Wulf and Lewthwaite 2016). This theory proposes that learning is enhanced through increased expectations mediated by the dopamine system. Consequently, heightened motivation predicts positive experiences, which can impact practice and learning. To our knowledge, to date, only two studies directly addressed this issue (Beik and Fazeli 2021; Beik et al. 2022), and none of them include differential learning. Accordingly, the question remains whether differential learning and contextual interference affect participants' motivation differently.

The objective of this study was to compare the effects of differential learning (DL) and contextual interference (CI)—each implemented at three levels (blocked, random, and progressive in the CI framework; predictable, stochastic, and progressive variation in the DL framework)—on motor skill acquisition and intrinsic motivation in novice basketball players. Specifically, this study examined how these practice structures influenced performance during acquisition, retention (fixed and variable), and transfer tests, as well as participants' motivation assessed through subscales of the Intrinsic Motivation Inventory (interest/enjoyment, perceived competence, and effort/importance). By integrating both performance and psychological measures, this study aimed to provide a comprehensive understanding of how variability‐based training methods affect motor learning and engagement in real‐world sport contexts.

Based on the explanations provided above, there is a lack of knowledge associated with comparing differential learning and contextual interference (using different levels, including blocked, random, and progressive in the CI framework and predictable, stochastic, and progressive in the DL framework), and also the effect of these two methods of practice on motivational aspects of the learning process is not well studied. Accordingly, based on the theoretical background (W. I. Schöllhorn et al. 2009), we hypothesized that differential learning would result in better performance during retention and transfer tests. In addition, based on the theoretical (Wulf and Lewthwaite 2016) and experimental (Beik and Fazeli 2021; Beik et al. 2022) background, we predicted that differential learning would result in higher intrinsic motivation than random or blocked practice. Previous research has shown that when the functional difficulty of a task aligns with the learner's skill level, both motor learning and intrinsic motivation are facilitated (Beik and Fazeli 2021; Beik et al. 2022). In line with this claim, empirical evidence suggests that progressive practice using the contextual interference framework—compared to blocked or random practice—can enhance learning (Porter and Magill 2010). This may be because progressive interference gradually raises task complexity, thereby avoiding the monotony often associated with blocked schedules and the early‐stage frustration linked to random practice. As a result, this approach may foster greater motivation. Furthermore, based on theoretical claims that differential learning introduces optimal noise (W. I. Schöllhorn et al. 2006) and empirical findings demonstrating its superiority over blocked and random practice (Mousavi et al. 2024), it is likely that differential learning increases motivation by providing task variability tailored to learners' skill levels. However, given the absence of direct empirical comparisons between progressive practice using the contextual interference framework and differential learning, and considering that both approaches are theorized to modulate practice variability in proportion to the learner's skill level, it is plausible that the motivational impact of differential learning may be comparable to that of progressive practice using the contextual interference framework. Additionally, it was predicted that moderate levels of interference (progressive schedule) and differential learning (progressive practice using the differential learning framework) would result in higher learning than the other extreme of these two training approaches. Building on the theoretical foundations and addressing the lack of practical comparisons between contextual interference and differential learning in relation to motor learning and motivational outcomes, the objective of this study was to compare the effects of differential learning (DL) and contextual interference (CI)—each implemented at three levels (blocked, random, and progressive using the CI framework and predictable, stochastic, and progressive variations using the DL framework)—on motor skill acquisition and intrinsic motivation in novice basketball players. Specifically, this study examined how these practice structures influenced performance (throwing accuracy) during acquisition, retention (fixed and variable), and transfer tests, as well as participants’ motivation assessed through subscales of the Intrinsic Motivation Inventory (interest/enjoyment, perceived competence, and effort/importance). By integrating both performance and psychological measures, this study aimed to provide a comprehensive understanding of how variability‐based training methods affect motor learning and engagement in real‐world sport contexts. Beyond theoretical significance, clarifying whether differential learning or contextual interference better supports skill transfer has direct implications for designing sports training protocols. For example, if differential learning enhances transfer (as hypothesized), coaches could prioritize movement variability (e.g., altering body positions) over task‐goal variability (e.g., changing shooting distances) when training novices in sports such as basketball. In addition, understanding motivational differences between methods could inform adherence strategies in youth sports programs, where intrinsic motivation predicts long‐term engagement. For rehabilitation contexts, identifying optimal “noise” levels could refine motor relearning protocols for clinical populations.

## 2 Materials and Methods

### 2.1 Participants

Novice (no prior formal basketball training) right‐handed (according to self‐report) female basketball players (84 students, mean age 16.2 ± 2.4) were asked to participate in this study and randomly divided into seven groups (*N* = 12). Although an a priori power analysis was not conducted before data collection, a post‐hoc power analysis was performed using G*Power 3.1. Assuming a medium effect size (*f* = 0.25), *α* = 0.05, and power = 0.80 for a repeated measures ANOVA (between‐within interaction), with 7 groups and 2 measurements, the required total sample size was estimated to be 63 participants. Our study included 84 participants (*n* = 12 per group), exceeding this threshold and ensuring adequate power to detect medium‐sized effects. Participants were randomly assigned to one of seven groups using a lottery method. Specifically, each group was assigned a unique number (1–7); numbers were written on identical slips of paper; slips were placed in an opaque bowl and thoroughly mixed; each participant drew one slip blindly to determine group assignment. All participants had normal or corrected‐to‐normal vision and had no neurological problem that affected motor control. A written informed consent form was obtained from all participants' parents, and they were allowed to leave the research process whenever they did not wish to continue. The study protocol was approved by the ethics committee of Shiraz University (code: SEP/14033/48/1059).

### 2.2 Task and Tools

Participants were asked to perform a basketball jump shot using a standard basketball hoop (height = 3.05 m) and ball (size 6, circumference = 72.5 cm, mass = 560 g). Each participant completed three consecutive days of practice, with three blocks per day and 18 trials per block, totaling 54 trials per session and 162 trials across all sessions. The total number of trials was equal for all experimental groups. To measure the throwing accuracy, a scoring system was used, which was based on previous studies (Wulf et al. 2005). The scoring system was defined as follows: a ball that went through the basket without touching the rim or the backboard scored 5 points; if the ball touched the ring or the backboard and then went through the basket, it scored 4 points; balls that only touched the ring (and did not go through the basket) scored 3 points; balls that touched both the backboard and the ring (and did not go through the basket) scored 2 points; balls that only touched the backboard (and did not go through the basket) received 1 point; if the ball touched neither the basket nor the backboard (air ball), 0 points were awarded. Additionally, to assess intrinsic motivation, the Intrinsic Motivation Inventory (IMI) was used (McAuley et al. 1989). The IMI was selected over alternative measures (e.g., Sport Motivation Scale, Behavioral Regulation in Exercise Questionnaire) for three key reasons. The first reason is theoretical alignment; the IMI's subscales (interest/enjoyment, perceived competence, effort/importance) directly map onto the psychological constructs central to OPTIMAL theory (Wulf and Lewthwaite 2016), which posits that intrinsic motivation enhances motor learning by strengthening goal‐action coupling. The second reason is validation in motor learning; the IMI has been empirically validated in sport and motor skill acquisition contexts (e.g., Badami et al. 2011), demonstrating high reliability (Cronbach's *α* > 0.80 for subscales) and sensitivity to practice schedule manipulations (Beik and Fazeli 2021). The third reason is practical utility; its brevity (9 items for our selected subscales) minimized participant burden while capturing multidimensional motivation, critical for detecting subtle differences between variability schedules (e.g., blocked vs. random practice). Three subscales, including interest/enjoyment, perceived competence, and effort/importance, were selected from the six subscales of the inventory. These subscales were chosen based on previous studies (Badami et al. 2011; Beik and Fazeli 2021), which showed their relevance in evaluating motivational aspects in motor learning tasks. These subscales were used to measure intrinsic motivation in previous studies (Badami et al. 2011; Beik and Fazeli 2021). The items used are shown in Table 1. Responses on the 7‐point Likert‐type scale ranged from 1 = strongly disagree to 7 = strongly agree. Negatively worded items were rescaled before data analysis.

**TABLE 1:** Items used from the Intrinsic Motivation Inventory (IMI) subscales in this study.

Subscale	Items
Interest/enjoyment	1. Basketball jump shot was fun to do. 2. While I was throwing the basketball, I was thinking about how much I enjoyed it. 3. I thought basketball jump shot was a boring activity. (R)
Perceived competence	4. After throwing the basketball for a while, I felt pretty competent. 5. I am satisfied with my basketball jump shot performance. 6. Basketball jump shot was an activity that I couldn't do very well. (R)
Effort/importance	7. I didn't try very hard to do well at basketball jump shot. (R) 8. It was important to me to do well at throwing the basketball. 9. I tried very hard while throwing the basketball

*Note:* (R) indicates reverse‐scored items.

### 2.3 Data Acquisition

**FIGURE 1:**
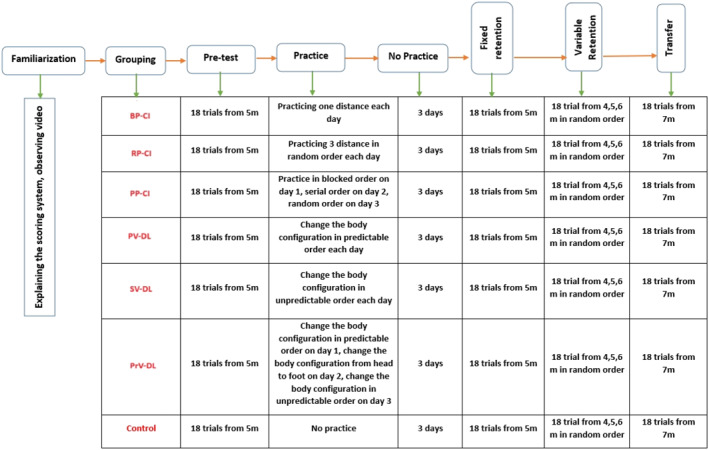
Schematic presentation of different phases of the experiment. BP‐CI, blocked practice–contextual interference framework; PP‐CI, progressive practice–contextual interference framework; PrV‐DL, progressive variation‐differential learning; PV‐DL, predictable variation‐differential learning; RP‐CI, random practice–contextual interference framework; SV‐DL, stochastic variation‐differential learning.

Figure 1 shows a schematic presentation of different phases of this experiment. After a short rest to become familiar with the experimental environment, the task and the experiment setup were explained to the participants. Nothing was told about the main goal of the experiment. The participants observed a video showing a skilled model (female player, 25 years old, 10 years of formal training) performing the jump shot task using a two‐handed technique from a distance of 5 m to the basket. The video was recorded from the sagittal plane, allowing for clear observation of the player, ball, and basket. Participants observed the video three times at 50% of normal speed (no verbal information was given to the participants). The scoring system for the basketball jump shot was also explained to the participants. Subsequently, the participant was randomly assigned to one of the seven groups. After a warm‐up process, including 5 min of running, rotating the upper main joints, rotating the lower main joints, and finally stretching movements (10 min in total), the subjects performed three attempts in order to familiarize themselves with the task. Then, a pretest including 18 trials from a 5‐m distance to the basket was performed to measure the initial accuracy of the participants. After the pretest, the participants practiced the jumping shot according to their grouping. In detail, the training protocol for each group was as follows.

#### 2.3.1 Random Practice–Contextual Interference Framework (RP‐CI)

Participants performed basketball jump shots randomly from three distances (4, 5, and 6 m) with the constraint that no distance was performed in two consecutive trials and each distance was repeated 6 times in each block.

#### 2.3.2 Blocked Practice–Contextual Interference Framework (BP‐CI)

In this group, participants engaged in practicing one distance per day. To reduce practice order effects, half of the participants in this group performed the task from the farthest distance to the nearest one, and the other half practiced from the nearest distance to the farthest one.

#### 2.3.3 Progressive Practice–Contextual Interference Framework (PP‐CI)

In this group, the training method comprised different approaches during each day. On the first day, participants engaged in a blocked schedule, where they practiced shooting at one distance in the first block, a different distance in the second block, and another different distance in the third block. The sequence of interval training on the first day followed the same counterbalanced approach as the BP‐CI group. On the second day, participants practiced with a serial schedule. Half of the participants performed a serial sequence where they practiced the first (4 m) to third (6 m) distance consecutively, repeating this serial six times within each block. The other half of the participants followed a serial sequence starting from the farthest distance and progressing to the nearest one. On the third day, participants performed the task using a random practice order, similar to the RP‐CI group.

#### 2.3.4 Stochastic Variation‐Differential Learning (SV‐DL)

In the SV‐DL group, participants experienced random changes in their body position every day, either in the upper‐ or lower‐body parts. These changes were introduced randomly, and all trials were different from each other in terms of movement variations. An example of the random order for differential learning instructions is as follows: Use your nondominant hand instead of your dominant hand for throwing the ball; start the movement with your left foot in front of your right foot; rotate your pelvis to the right, then make the throw; throw the ball with only one hand instead of using both hands; start the movement with your right foot in front of your left foot; rotate your pelvis to the left, then make the throw; keep your hands completely tense while throwing and throw the ball with your right hand; lean your torso forward, then make the throw; stand in a half‐squat position, then make the throw; bend your torso to the right, then make the throw; keep your hands completely tense while throwing and throw the ball with your left hand; bend your torso to the left, then make the throw; lean your torso backward, then make the throw; contract your right leg muscles, then make the throw; keep your hands completely relaxed while throwing and throw the ball with your right hand; contract your left leg muscles, then make the throw; keep your hands completely relaxed while throwing and throw the ball with your left hand; contract the muscles of both legs, then make the throw.

#### 2.3.5 Predictable Variation‐Differential Learning (PV‐DL)

Participants in this group practiced the basketball jump shot with systematic changes in body configuration introduced in a predictable (blocked) order within each session. For example, one set of changes targeted the upper extremities (e.g., throwing with the nondominant hand), followed by changes in the trunk, and then the lower extremities. The same predictable sequence of variations was repeated across all three blocks of a day. Half of the participants progressed from lower‐ to upper‐body changes, whereas the other half followed the reverse order. Importantly, no randomization or serial alternation of body configurations occurred within or across blocks on the same day. The blocked order for the abovementioned examples of differential learning instructions would be as follows: Use your nondominant hand instead of your dominant hand for throwing the ball; throw the ball with only one hand instead of using both hands; keep your hands completely tense while throwing and throw the ball with your right hand; keep your hands completely tense while throwing and throw the ball with your left hand; keep your hands completely relaxed while throwing and throw the ball with your right hand; keep your hands completely relaxed while throwing and throw the ball with your left hand; rotate your pelvis to the right, then make the throw; rotate your pelvis to the left, then make the throw; lean your torso forward, then make the throw; lean your torso backward, then make the throw; bend your torso to the right, then make the throw; bend your torso to the left, then make the throw; start the movement with your right foot in front of your left foot; start the movement with your left foot in front of your right foot; stand in a half‐squat position, then make the throw; contract your right leg muscles, then make the throw; contract your left leg muscles, then make the throw; contract the muscles of both legs, then make the throw.

#### 2.3.6 Progressive Variation‐Differential Learning (PrV‐DL)

On the first day, participants experienced changes in a blocked manner, similar to the PV‐DL group. On the second day, the changes were introduced sequentially, either from the top to the bottom of the body (for half of the participants) or from the bottom to the top (for the other half). On the third day, the order of implementing these changes was randomized, ensuring a variable and unpredictable order. An example of progressive variation using the differential learning method is as follows.

##### 2.3.6.1 First Day

Use your nondominant hand instead of your dominant hand for throwing the ball; throw the ball with only one hand instead of using both hands; lean your torso forward, then make the throw; lean your torso backward, then make the throw; start the movement with your right foot in front of your left foot; start the movement with your left foot in front of your right foot.

##### 2.3.6.2 Second Day

Keep your hands completely tense while throwing and throw the ball with your right hand; lean your torso forward, then make the throw; stand in a half‐squat position, then make the throw; keep your hands completely tense while throwing and throw the ball with your left hand; lean your torso backward, then make the throw; contract your right leg muscles, then make the throw.

##### 2.3.6.3 Third Day

Bend your torso to the right, then make the throw; keep your hands completely relaxed while throwing and throw the ball with your right hand; contract the muscles of both legs, then make the throw; keep your hands completely relaxed while throwing and throw the ball with your left hand; contract your left leg muscles, then make the throw; bend your torso to the left, then make the throw.

It is essential to note that during each day of training, participants completed three blocks of 18 trials. The aforementioned instructions serve merely as illustrative examples for comprehending the development of differential instruction in stochastic, predictable, and progressive formats (the number of trials each day was equal between different groups). All experimental groups engaged in the differential learning trials performed their training at a distance of 5 m from the basket, with adjustments restricted exclusively to variations in body positioning throughout the training sessions. In all DL conditions (SV‐DL, PV‐DL, PrV‐DL), each of the 162 practice trials had a unique movement variation; no instruction was repeated during the entire practice period. The distinction between DL groups lay solely in the sequential logic of introducing these variations. Participants were not expected to memorize any set of instructions. Instead, the experimenter verbally provided the specific instruction immediately before each trial and, when necessary, gave a brief physical demonstration. This ensured that participants, all of whom were novices, could easily understand and apply the instruction for that trial without having to recall previous instructions.

#### 2.3.7 Control

Participants did not practice the task and just performed the tests.

On the third day of training, and after the final block, participants completed an intrinsic motivation questionnaire to assess their motivation levels (as the control group did not perform any practice trial, the participants in this group did not complete this questionnaire).

Three days after the final training session, participants completed two retention tests. One of these tests was similar to the pretest (constant retention test), and the other test included throwing from different distances (4, 5, and 6 m) in a random order (variable retention test). The order of these two tests was counterbalanced. Additionally, after these retention tests, a transfer test was performed from a 7‐m distance to the basket.

### 2.4 Data Analysis

The mean scores for each day of practice and each test were calculated as the accuracy measure. To compare the performance of the experimental groups during acquisition, practice data were analyzed in a 6 (groups; RP‐CI, BP‐CI, PP‐CI, SV‐DL, PV‐DL, and PrV‐DL) × 3 (block, days of practice) mixed‐design ANOVA with repeated measures on the last factor. It is important to note that the control group was excluded from this analysis, as it did not practice the task. To analyze the fixed retention test, the data of the pretest were also included to show the improvement from pretest to retention test. The data were analyzed using a 7 (groups) × 2 (block, pretest and fixed retention test) mixed‐design ANOVA with repeated measures on the last factor. Additionally, to analyze the variable retention test and the transfer test, separate one‐way ANOVAs were used. In addition, to analyze the data related to the subscales of the motivation questionnaire, three separate one‐way ANOVAs were used. All analyses used an alpha level of 0.05 for significance thresholds. Effect sizes were interpreted using $\eta_{p}^{2}$ (partial eta‐squared) with thresholds of 0.01 (small), 0.06 (medium), and 0.14 (large) for ANOVA effects (Cohen 1988). Post‐hoc comparisons employed the LSD tests in all significant main/interaction effects reported in results.

## 3 Results

### 3.1 Acquisition

**FIGURE 2:**
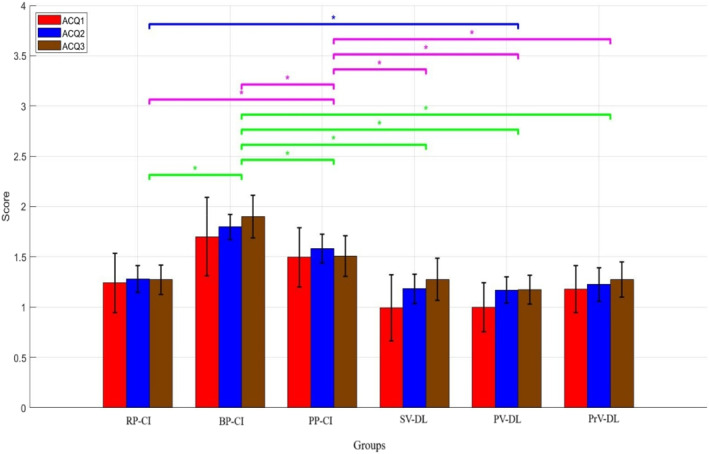
The accuracy of different groups during the acquisition. Error bars represent the SD. ACQ1, first day of acquisition; ACQ2, second day of acquisition; ACQ3, third day of acquisition; BP‐CI, blocked practice–contextual interference framework; PP‐CI, progressive practice–contextual interference framework; PrV‐DL, progressive variation‐differential learning; PV‐DL, predictable variation‐differential learning; RP‐CI, random practice–contextual interference framework; SD, standard deviation; SV‐DL, stochastic variation‐differential learning.

Figure 2 shows the accuracy performance of all groups during the acquisition. For the acquisition data, as the Mauchly's test of sphericity was violated, the Greenhouse–Geisser adjustment was used. The results of ANOVA for the acquisition data revealed significant main effects for group (*F*_(5,66)_ = 30.49; *p* < 0.001, $\eta_{p}^{2}$ = 0.69) and block (*F*_(1.53,100.99)_ = 11.36, *p* < 0.001, $\eta_{p}^{2}$ = 0.14). However, the interaction effect between group and block was not significant (*F*_(7.65,100.99)_ = 1.27; *p* = 0.25, $\eta_{p}^{2}$ = 0.08).

The results of the LSD post‐hoc test for the group effect showed that the BP‐CI group exhibited significantly higher accuracy compared to all other groups (all *p* < 0.05). Additionally, the PP‐CI group demonstrated significantly different performance compared to all other groups (all *p* < 0.05). Additionally, the difference between the RP‐CI and PV‐DL groups was significant (*p* < 0.05). No significant differences were found among the other groups (all *p* > 0.05). Comparing the mean values indicated that the BP‐CI group and the PP‐CI group achieved higher accuracy than the other groups (means: RP‐CI = 1.26, BP‐CI = 1.79, PP‐CI = 1.52, SV‐DL = 1.15, PV‐DL = 1.11, PrV‐DL = 1.22). As could be seen from the results, the differential groups did not perform more accurately than other groups during the acquisition.

The LSD post‐hoc test for the main effect of block showed that the accuracy on the first day of the acquisition was significantly different from the accuracy on the subsequent 2 days (all *p* < 0.05). However, no significant difference was found between the accuracy on the second and third days (*p* > 0.05). The comparison of the means indicated that the groups exhibited higher accuracy on the second and third days compared to the first day (means: first day = 1.26, second day = 1.37, third day = 1.40).

### 3.2 Retention Tests

#### 3.2.1 Fixed Retention Test

**FIGURE 3:**
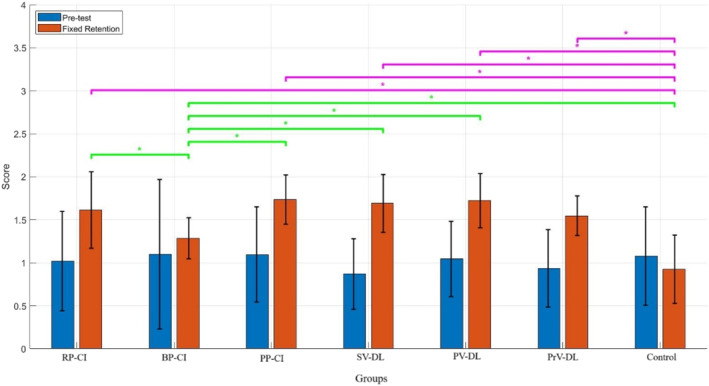
The accuracy of different groups during the pretest and fixed retention test. Error bars represent the SD. BP‐CI, blocked practice–contextual interference framework; PP‐CI, progressive practice–contextual interference framework; PrV‐DL, progressive variation‐differential learning; PV‐DL, predictable variation‐differential learning; RP‐CI, random practice–contextual interference framework; SD, standard deviation; SV‐DL, stochastic variation‐differential learning.

Figure 3 shows the accuracy performance of all groups during the pretest and the fixed retention test. The results of mixed ANOVA did not show a significant main effect of group (*F*_(6,77)_ = 1.72; *p* = 0.127, $\eta_{p}^{2}$ = 0.11). However, the main effect of block (*F*_(1,77)_ = 58.19; *p* < 0.001, $\eta_{p}^{2}$ = 0.43) and the interaction effect between group and block (*F*_(6,77)_ = 4.15; *p* = 0.001, $\eta_{p}^{2}$ = 0.24) were significant. A post‐hoc test was performed for the interaction effect. The results showed no significant difference between groups during the pretest (all *p* > 0.05). However, significant differences were observed between groups during the retention test. Although, during the retention test, the difference between the BP‐CI group and the PrV‐DL group was not significant, the differences between the BP‐CI group and all other groups were significant (all *p* < 0.05). Additionally, all groups demonstrated a significant difference compared to the control group (all *p* < 0.05). Comparing the mean values indicated the lower performance of the BP‐CI group and the control group (means: RP‐CI = 1.61, BP‐CI = 1.28, PP‐CI = 1.73, SV‐DL = 1.69, PV‐DL = 1.72, PrV‐DL = 1.54, control = 0.92). As could be seen from the results, the differential groups (except for the PrV‐DL group) performed more accurately than the BP‐CI group during this test.

In addition, the results of the post‐hoc test revealed significant improvements for all experimental groups (all *p* < 0.05), from the pretest to the retention test except for the control group (*p* > 0.05). Comparing the means showed that experimental groups performed more accurately during the retention than the pretest (means: RP‐CI pretest = 1.02, RP‐CI retention = 1.61, BP‐CI pretest = 1.098, BP‐CI retention = 1.28, PP‐CI pretest = 1.095, PP‐CI retention = 1.73, SV‐DL pretest = 0.87, SV‐DL retention = 1.69, PV‐DL pretest = 1.044, PV‐DL retention = 1.72, PrV‐DL pretest = 0.93, PrV‐DL retention = 1.54, control pretest = 1.07, control retention = 0.92).

#### 3.2.2 Variable Retention

**FIGURE 4:**
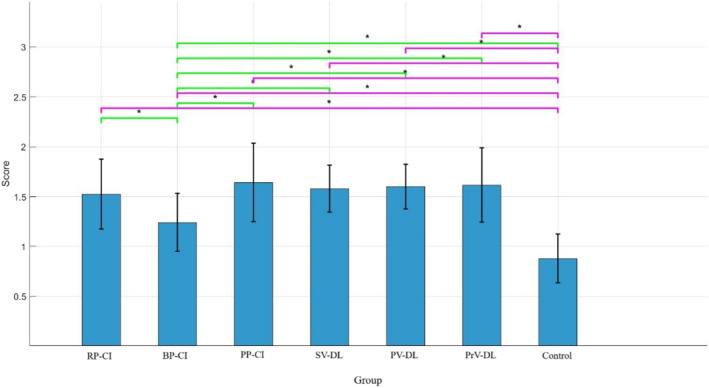
The accuracy of different groups during the variable retention test. Error bars represent the SD. BP‐CI, blocked practice–contextual interference framework; PP‐CI, progressive practice–contextual interference framework; PrV‐DL, progressive variation‐differential learning; PV‐DL, predictable variation‐differential learning; RP‐CI, random practice–contextual interference framework; SD, standard deviation; SV‐DL, stochastic variation‐differential learning.

Figure 4 shows the accuracy performance of all groups during the variable retention test. The results of ANOVA for the variable retention test indicated a significant main effect of group (*F*_(6,83)_ = 22.04, *p* < 0.001). The LSD post‐hoc test showed that the BP‐CI group performed significantly differently from the other groups (all *p* < 0.05). It was also observed that all experimental groups were significantly different from the control group (all *p* < 0.05). However, no significant differences were found among the other experimental groups (all *p* > 0.05). Comparing the mean values indicated that the BP‐CI group and the control group had lower performance compared to the other groups (means: RP‐CI = 1.52, BP‐CI = 1.24, PP‐CI = 1.64, SV‐DL = 1.57, PV‐DL = 1.60, PrV‐DL = 1.61, control = 0.88). As could be seen from the results, the differential learning groups performed more accurately than the BP‐CI group during this test.

#### 3.2.3 Transfer Test

**FIGURE 5:**
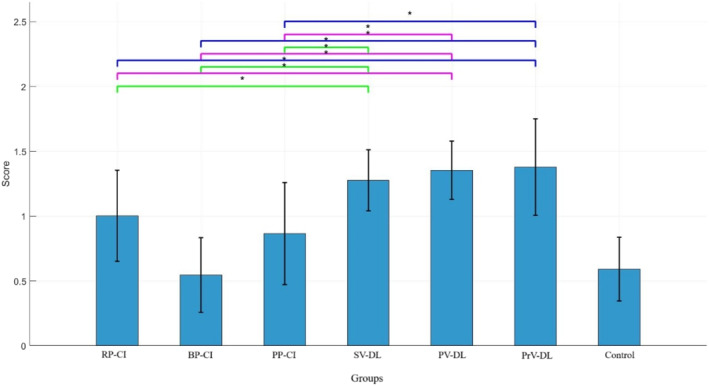
The accuracy of different groups during the transfer test. Only the significant differences between differential groups and contextual interference groups are shown. Error bars represent the SD. BP‐CI, blocked practice–contextual interference framework; PP‐CI, progressive practice–contextual interference framework; PrV‐DL, progressive variation‐differential learning; PV‐DL, predictable variation‐differential learning; RP‐CI, random practice–contextual interference framework; SD, standard deviation; SV‐DL, stochastic variation‐differential learning.

Figure 5 shows the accuracy performance of all groups during the transfer test. The results of ANOVA showed a significant main effect of group (*F*_(6,83)_ = 15.50; *p* < 0.001). The post‐hoc test (LSD) revealed that the BP‐CI group performed significantly differently from all other groups (all *p* < 0.05) except the control group (*p* > 0.05). Additionally, the RP‐CI group and PP‐CI group showed significant differences compared to the other experimental groups and the control group (all *p* < 0.05). Furthermore, although the differential learning groups did not significantly differ from each other (all *p* > 0.05), they exhibited significant differences compared to other groups, including the control group (all *p* < 0.05).

Group mean comparisons revealed that the differential learning groups performed better than the other groups during the transfer test. Moreover, the RP‐CI group and PP‐CI group demonstrated higher accuracy compared to the BP‐CI group (means: RP‐CI = 1.002, BP‐CI = 0.54, PP‐CI = 0.86, SV‐DL = 1.27, PV‐DL = 1.35, PrV‐DL = 1.38, control = 0.59).

### 3.3 Intrinsic Motivation Subscales

#### 3.3.1 Interest/Enjoyment

**FIGURE 6:**
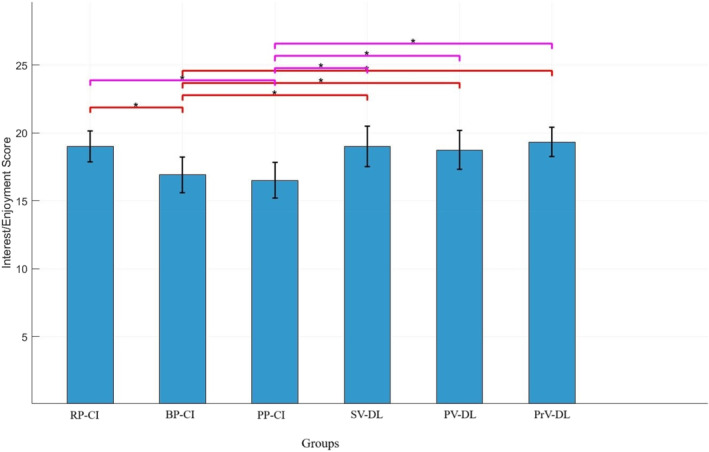
Scores for the interest/enjoyment subscale of the intrinsic motivation questionnaire. Error bars represent the SD. BP‐CI, blocked practice–contextual interference framework; PP‐CI, progressive practice–contextual interference framework; PrV‐DL, progressive variation‐differential learning; PV‐DL, predictable variation‐differential learning; RP‐CI, random practice–contextual interference framework; SD, standard deviation; SV‐DL, stochastic variation‐differential learning.

Figure 6 shows the scores for the interest/enjoyment subscale. The results of ANOVA showed a significant main effect of group (*F*_(5,71)_ = 10.56, *p* < 0.001). The post‐hoc test (LSD) revealed that the BP‐CI group was significantly different from all other groups (all *p* < 0.05) except for the PP‐CI group (*p* > 0.05). Similarly, the differences between the PP‐CI group and other experimental groups were significant (all *p* < 0.05). A comparison of the mean scores showed that the RP‐CI and all differential groups obtained higher scores in this subscale compared to the BP‐CI and PP‐CI groups (means: RP‐CI = 19, BP‐CI = 16.91, PP‐CI = 16.5, SV‐DL = 19, PV‐DL = 18.75, PrV‐DL = 19.33).

#### 3.3.2 Perceived Competence

**FIGURE 7:**
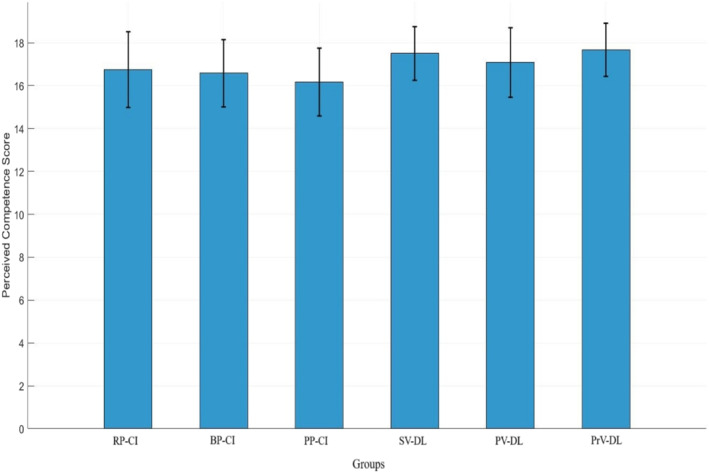
Scores for the perceived competence subscale of the intrinsic motivation questionnaire. Error bars represent the SD. BP‐CI, blocked practice–contextual interference framework; PP‐CI, progressive practice–contextual interference framework; PrV‐DL, progressive variation‐differential learning; PV‐DL, predictable variation‐differential learning; RP‐CI, random practice–contextual interference framework; SD, standard deviation; SV‐DL, stochastic variation‐differential learning.

Figure 7 shows the scores of the perceived competence subscale. The results showed no significant difference between the groups in this subscale (*F*_(5,71)_ = 1.69, *p* > 0.05).

#### 3.3.3 Effort/Importance

**FIGURE 8:**
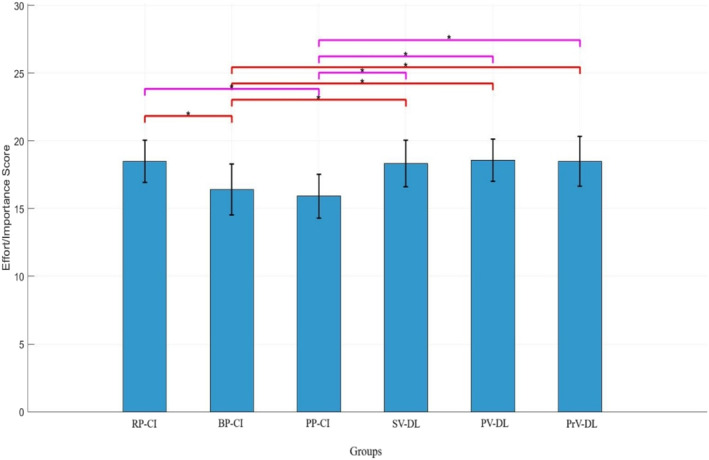
Scores for the effort/importance subscale of the intrinsic motivation questionnaire. BP‐CI, blocked practice–contextual interference framework; PP‐CI, progressive practice–contextual interference framework; PrV‐DL, progressive variation‐differential learning; PV‐DL, predictable variation‐differential learning; RP‐CI, random practice–contextual interference framework; SD, standard deviation; SV‐DL, stochastic variation‐differential learning.

Figure 8 shows the scores related to the effort/importance subscale. The results of ANOVA showed a significant difference between groups in this subscale (*F*_(5,71)_ = 6.03, *p* < 0.001). The results of the LSD post‐hoc test showed that the BP‐CI and PP‐CI groups did not perform significantly differently from each other (*p* > 0.05); however, these two groups performed significantly differently from other experimental groups (all *p* < 0.05). No other significant effect was observed (all *p* > 0.05). Means comparison indicated higher scores for the RP‐CI group and all differential groups (means: RP‐CI = 18.5, BP‐CI = 16.41, PP‐CI = 15.91, SV‐DL = 18.33, PV‐DL = 18.58, PrV‐DL = 18.5).

## 4 Discussion

The purpose of this study was to examine the effects of different levels of contextual interference and differential learning on learning basketball jump shots and participants’ motivation. The results revealed that during the acquisition phase, the BP‐CI group outperformed the other groups, whereas during the retention tests (constant and variable tests), it exhibited weaker performance compared to the RP‐CI group and other groups. Furthermore, during the transfer test, the RP‐CI and PP‐CI groups exhibited higher accuracy than the BP‐CI group. These findings are consistent with previous research associated with the contextual interference effect. In previous studies, two effects of contextual interference have been reported. The first effect suggests that the blocked schedule would result in higher performance than the random schedule during the acquisition phase, whereas the second effect indicates that the random schedule would perform better than the blocked schedule during the retention or transfer tests (Shea and Morgan 1979; Wright and Kim 2019). These results could be explained using different perspectives. On the one hand, the formation of a stronger mental representation during random practice could be the possible reason for this superior performance, as proposed by the elaboration and distinctiveness processing hypothesis (Shea and Morgan 1979) and the forgetting and reconstitution hypothesis (Lee and Magill 1983). On the other hand, it is argued that introducing changes using different distances leads to movement variability, which, in turn, facilitates exploration of the solution space and guides performers toward learning an optimal pattern. This argument aligns with the findings of differential learning (W. I. Schöllhorn et al. 2009). A novel perspective on the use of noise in training suggests that different training approaches inherently contain varying levels of noise. According to this view, repetitive training includes the lowest level of noise, followed by a methodical series of exercises with higher noise levels than repetitive training. Variability of practice, contextual interference, and differential learning are ranked successively higher in terms of noise levels (W. I. Schöllhorn et al. 2009). From this perspective, differential learning represents an optimal level of noise for learning. This argument is supported in this study by the findings related to the differential learning groups.

The results demonstrated that the differential groups (SV‐DL, PV‐DL, and PrV‐DL) did not show significant differences compared to the contextual interference groups (RP‐CI and PP‐CI) in the fixed and variable retention tests. The possible reason for this finding could be the duration of the training in this study. Other studies that employed longer training durations reported more positive results (Oftadeh et al. 2021; W. I. Schöllhorn et al. 2006). Training duration has also been identified as an important factor in the contextual interference effect (Boutin and Blandin 2010). Thus, it appears that, in addition to the type of noise in training, the duration of noise application is a significant factor influencing the learning process. Retention tasks, being similar to the acquisition conditions, may require more extended exposure for between‐group differences to emerge. In contrast, transfer tasks involve novel performance conditions, which may allow the benefits of DL—particularly the greater movement variability—to become apparent even after a short training period. Previous DL and CI studies have often used longer training durations: W. I. Schöllhorn et al. (2006) implemented 12 practice sessions, Oftadeh et al. (2021) used 24 sessions, and Apidogo et al. (2022) used 18 sessions. Compared to these, our 3‐session schedule may have been insufficient for retention differences to reach statistical significance. However, the same duration was enough to reveal significant advantages for DL in the transfer test, likely because transfer conditions more directly benefit from the enhanced adaptability fostered by higher practice variability.

During the transfer test, the differential groups (SV‐DL, PV‐DL, and PrV‐DL) had higher accuracy than the contextual interference groups (RP‐CI, BP‐CI, and PP‐CI). These findings are consistent with previous studies that highlight the superiority of differential learning over contextual interference for learning motor skills (Apidogo et al. 2022). Differential learning, derived from the dynamical systems perspective and artificial neural networks, considers learning as an aspect of the living system that has often been overlooked. The concept of stochastic resonance, which serves as the foundation of differential learning, refers to relative adjustments between two stochastic signals. One signal represents internal noise originating from the learner, whereas the other represents external noise arising from different levels of changes inherent in the practice approach (W. I. Schöllhorn et al. 2006). Optimal adjustment of external noise compared to internal noise is crucial for motor learning improvement. Deviating from the optimal level of adjustments, whether higher or lower, will negatively affect learning (Moss et al. 2004). According to this view, the optimal level of noise is achieved in the differential learning approach, whereas contextual interference provides a lower level of noise that may not properly stimulate the exploration of solution space and self‐organization (W. I. Schöllhorn et al. 2009).

Although previous perspectives considered movement fluctuation as a detrimental factor (R. A. Schmidt et al. 1979) that should be minimized to enhance learning, the dynamic systems perspective suggests that increased fluctuations would be necessary for phase transition, supporting self‐organization (Schöner and Kelso 1988). Increased variability is considered an unstable state that requires less effort to change compared to starting from a stable state (W. I. Schöllhorn 2016). Therefore, increasing variability and movement fluctuations facilitate the exploration of the solution space, helping individuals find a pattern that fits their characteristics. It is possible that changing body position in every trial facilitated the process of self‐organization and finding a movement solution optimal for the learner. Accordingly, the noise provided by changing the body position may have a more beneficial effect than the noise provided by changing the distance from the target.

Similar principles are supported in artificial neural network research. Research on artificial neural networks demonstrates that training with noisy data leads to more robust network applications (Haykin 1994). When all data are very similar, the network's learning performance is compromised, and it becomes unable to interpret data with deviations (Haykin 1994). Training artificial networks with noisy data expands the solution space, enabling interpolation during the application phase. In contrast to variability of practice and contextual interference, which aim to repeat an optimal movement pattern, differential learning considers noise‐like fluctuations in the movement pattern as necessary for self‐organization. It does not accept the concept of an optimal movement pattern for all learners. Based on these explanations, the presence of optimal noise during training may have facilitated a more effective exploration of the solution space and supported self‐organization, ultimately contributing to the superior performance of the differential groups during the transfer test. Conversely, the presence of nonoptimal noise in the contextual interference conditions could explain the poorer performance of these groups during the transfer test.

Although our transfer‐test findings align with the view that DL can facilitate exploration of the solution space, we temper this conclusion in light of critical evaluations. Künzell and Hossner (2012, 2013), Hossner et al. (2016a, 2016b), and Tassignon et al. (2021) caution that without standardized definitions of “noise” and without consistent replication, DL advantages may be task‐specific or driven by transient increases in arousal rather than genuine self‐organization processes. Future research ought to employ larger, multisite samples, pre‐registered protocols, and clearly operationalized noise manipulations to determine under which conditions—if any—DL yields reliably superior retention and transfer.

These findings could be explained according to another view. It is believed that variability can be utilized at different levels, task goal, and movement redundancy (Ranganathan and Newell 2013). The contextual interference and variability of practice use different task goals to enhance learning, whereas differential learning uses variability at the level of movement redundancy. Although previous studies indicated higher motor learning as the result of variability at the level of task goals (Ranganathan and Newell 2010), the results of this study were in favor of variability at the level of movement redundancy. Changing body configuration using differential learning protocol resulted in equal performance to random practice during the retention tests and higher performance during the transfer test than all contextual interference groups (RP‐CI, BP‐CI, and PP‐CI). It seems that inserting noise into practice using variability at the level of redundancy resulted in a more optimal level of noise that facilitates learning of the task.

Additionally, the results of this study indicated that the differential groups (SV‐DL, PV‐DL, and PrV‐DL) and the RP‐CI group exhibited higher levels of internal motivation (the subscales of interest/enjoyment and effort/importance) compared to the other groups. These findings align partially with previous research demonstrating the impact of training methods on learners' motivation (Beik and Fazeli 2021; Beik et al. 2022). It appears that the presence of noise in the motor system can enhance the learner's interest in the task by introducing changes during practice. This higher motivation may encourage exploration behavior and facilitate the process of self‐organization. Previous studies indicated higher internal motivation during practice when the level of noise changes in accordance with improvement of learner skill level, according to a specific error level (Beik and Fazeli 2021; Beik et al. 2022). In line with this argument, the theory of optimal learning (Wulf and Lewthwaite 2016) suggests that motivation serves as an important factor for motor learning. However, it is worth noting that despite the significant performance difference between the random practice using the contextual interference framework (RP‐CI group) and the differential learning groups (SV‐DL, PV‐DL, and PrV‐DL) in the transfer test, no difference was observed in their levels of motivation. Although the RP‐CI group outperformed the BP‐CI group during the retention, it performed worse than the differential groups (SV‐DL, PV‐DL, and PrV‐DL) during the transfer test. This supports the importance of the methods of providing noise during practice. Another possible explanation could be attributed to the sensitivity of measuring intrinsic motivation in our study. Previous research has suggested using more objective methods such as EEG to measure internal motivation, which could serve as a more sensitive measure in future studies. As a previous study showed, internal motivation may change with the progress of practice (Beik et al. 2022). Random practice may result in higher motivation at the end of practice, when the learner acquires an acceptable level of accuracy. Accordingly, our method of measurement could not show such changes in the motivation level in different groups.

These findings have direct implications for basketball skill acquisition. For instance, the superior transfer performance observed in the differential learning groups (SV‐DL, PV‐DL, and PrV‐DL) suggests that introducing variability in body posture—such as shooting from a half‐squat or with altered arm positions—may help players adapt to unpredictable game scenarios, such as shooting under defensive pressure or from off‐balance positions. Similarly, the enhanced motivation in these groups could reflect the engaging nature of constantly changing movement challenges, which may prevent monotony and sustain interest during repetitive drills. In contrast, the BP‐CI group, despite showing higher accuracy during acquisition, may have developed rigid movement patterns that are less adaptable during dynamic in‐game situations, such as fast breaks or contested shots. These results highlight the importance of designing training that not only improves performance in controlled settings but also prepares athletes for the variability and complexity of real gameplay.

## 5 Conclusions and Limitations

According to the results of this study, differential learning results in motor learning that is more transferable than contextual interference. Additionally, using a high level of noise would result in higher motivation. These findings were interpreted according to the role of noise in exploration and self‐organization. It has been argued that differential learning enhances exploration behavior and leads to self‐organization. Although random practice may also induce some exploration behavior, the noise level in differential learning is likely optimal for provoking exploration within the solution space. Consequently, the differential learning groups (SV‐DL, PV‐DL, and PrV‐DL) demonstrated greater accuracy than the random practice group (RP‐CI) during the transfer test.

Like any other study, our study also has limitations. We did not measure the movement patterns and their variability as a result of the practice method. Because an action can be performed using different movement patterns that yield similar outcomes, it is recommended that future studies measure the variability in movement patterns as the result of different practice methods. Additionally, because random practice using the contextual interference framework (RP‐CI) showed beneficial effects during the retention tests, one recommendation could be to combine two types of practice—differential learning and random practice using the contextual interference framework—within a single paradigm to evaluate their potential effects. Additionally, we did not measure participants' motivation before the experiment. Therefore, a research design that includes measuring internal motivation both before and after the experiment could be beneficial. Additionally, as we did not measure motivation and movement variability before and during the practice, we do not specifically know if variability increases motivation, which, in turn, increases learning, or if variability directly increases learning, or if increased motivation leads to more variability (exploration), which then enhances learning. Accordingly, future studies can compare different practice schedules in terms of internal motivation and movement variations (variability at the kinematics level) they provide during practice, as it is argued that higher noise levels might result in higher variation, encouraging exploration of the solution space and self‐organization. Finally, because there were no significant differences between the differential learning groups (SV‐DL, PV‐DL, and PrV‐DL) and the high‐interference groups (random and progressive schedules; RP‐CI and PP‐CI groups) during the retention tests—interpreted as due to the short training duration—it is recommended that future studies incorporate longer practice periods.

## Conflicts of Interest

The authors declare no conflicts of interest.

## Data Availability

The data that support the findings of this study are available from the authors and have been uploaded on a digital database (https://doi.org/10.5281/zenodo.10377112) but restrictions apply to the availability of these data by Shiraz University, and so they are not publicly available. Data are, however, available from the authors upon reasonable request and with permission from Shiraz University.

## References

[ejsc70061-bib-0001] Apidogo, J. B. , J. Burdack , and W. I. Schöllhorn . 2022. “Learning Multiple Movements in Parallel—Accurately and in Random Order, or Each With Added Noise?” International Journal of Environmental Research and Public Health 19, no. 17: 10960. 10.3390/ijerph191710960. 36078674 PMC9517918

[ejsc70061-bib-0002] Badami, R. , M. VaezMousavi , G. Wulf , and M. Namazizadeh . 2011. “Feedback After Good Versus Poor Trials Affects Intrinsic Motivation.” Research Quarterly for Exercise & Sport 82, no. 2: 360–364. 10.5641/027013611x13119541884347. 21699117

[ejsc70061-bib-0003] Beckmann, H. , C. Winkel , and W. I. Schöllhorn . 2010. “Optimal Range of Variation in Hockey Technique Training.” International Journal of Sport Psychology 41, no. 4: 5–45.

[ejsc70061-bib-0004] Beik, M. , and D. Fazeli . 2021. “The Effect of Learner‐Adapted Practice Schedule and Task Similarity on Motivation and Motor Learning in Older Adults.” Psychology of Sport and Exercise 54: 101911. 10.1016/j.psychsport.2021.101911.

[ejsc70061-bib-0005] Beik, M. , H. Taheri , A. S. Kakhki , M. Ghoshuni , and D. Fazeli . 2022. “Contextual Interference Effects on Approach Motivation When Learning Timing Tasks: A Frontal Electroencephalography (EEG) Alpha Asymmetry Study in Older Adults.” Perceptual and Motor Skills 129, no. 4: 1321–1341. 10.1177/00315125221098325. 35511777

[ejsc70061-bib-0006] Boutin, A. , and Y. Blandin . 2010. “Cognitive Underpinnings of Contextual Interference During Motor Learning.” Acta Psychologica 135, no. 2: 233–239. 10.1016/j.actpsy.2010.07.004. 20684941

[ejsc70061-bib-0008] Brady, F. 2008. “The Contextual Interference Effect and Sport Skills.” Perceptual and Motor Skills 106, no. 2: 461–472. 10.2466/pms.106.2.461-472. 18556902

[ejsc70061-bib-0009] Buszard, T. , M. Reid , L. Krause , S. Kovalchik , and D. Farrow . 2017. “Quantifying Contextual Interference and Its Effect on Skill Transfer in Skilled Youth Tennis Players.” Frontiers in Psychology 8: 1931. 10.3389/fpsyg.2017.01931. 29163306 PMC5676081

[ejsc70061-bib-0010] Cohen, J. 1988. Statistical Power Analysis for the Behavioral Sciences. L. Erlbaum Associates.

[ejsc70061-bib-0011] Davids, K. , S. Bennett , and K. M. Newell . 2006. Movement System Variability. Human Kinetics.

[ejsc70061-bib-0012] Farrow, D. , and T. Buszard . 2017. “Exploring the Applicability of the Contextual Interference Effect in Sports Practice.” Progress in Brain Research 234: 69–83. 10.1016/bs.pbr.2017.07.002. 29031473

[ejsc70061-bib-0013] Guadagnoli, M. A. , and T. D. Lee . 2004. “Challenge Point: A Framework for Conceptualizing the Effects of Various Practice Conditions in Motor Learning.” Journal of Motor Behavior 36, no. 2: 212–224. 10.3200/jmbr.36.2.212-224. 15130871

[ejsc70061-bib-0014] Haykin, S. 1994. Neural Networks: A Comprehensive Foundation. MacMillan College Publishing Co.

[ejsc70061-bib-0015] Henz, D. , A. John , C. Merz , and W. I. Schöllhorn . 2018. “Post‐Task Effects on EEG Brain Activity Differ for Various Differential Learning and Contextual Interference Protocols.” Frontiers in Human Neuroscience 12: 19. 10.3389/fnhum.2018.00019. 29445334 PMC5797795

[ejsc70061-bib-0016] Hossner, E.‐J. , B. Käch , and J. Enz . 2016a. “On the Optimal Degree of Fluctuations in Practice for Motor Learning.” Human Movement Science 47: 231–239. 10.1016/j.humov.2015.06.007. 26123921

[ejsc70061-bib-0017] Hossner, E.‐J. , B. Käch , and J. Enz . 2016b. “On Experimental Designs, Differencial Learning, Theoretical Issues, Dynamical Systems, and the Capability to Adapt: Response to Schöllhorn.” Human Movement Science 47: 246–249. 10.1016/j.humov.2015.11.019. 26705908

[ejsc70061-bib-0019] Künzell, S. , and E. J. Hossner . 2012. “Differenzielles Lehren und Lernen: eine Kritik.” Sportwissenschaft 42, no. 2: 83–95. 10.1007/s12662-012-0251-y.

[ejsc70061-bib-0020] Künzell, S. , and E.‐J. Hossner . 2013. “Differenzielles Lehren und Lernen.” Sportwissenschaft 43, no. 1: 61–62. 10.1007/s12662-013-0287-7.

[ejsc70061-bib-0021] Lee, T. D. , and R. A. Magill . 1983. “The Locus of Contextual Interference in Motor‐Skill Acquisition.” Journal of Experimental Psychology: Learning, Memory, and Cognition 9, no. 4: 730–746. 10.1037/0278-7393.9.4.730.

[ejsc70061-bib-0022] Magill, R. A. , and K. G. Hall . 1990. “A Review of the Contextual Interference Effect in Motor Skill Acquisition.” Human Movement Science 9, no. 3–5: 241–289. 10.1016/0167-9457(90)90005-x.

[ejsc70061-bib-0023] McAuley, E. , T. Duncan , and V. V. Tammen . 1989. “Psychometric Properties of the Intrinsic Motivation Inventory in a Competitive Sport Setting: A Confirmatory Factor Analysis.” Research Quarterly for Exercise & Sport 60, no. 1: 48–58. 10.1080/02701367.1989.10607413. 2489825

[ejsc70061-bib-0024] Moss, F. , L. M. Ward , and W. G. Sannita . 2004. “Stochastic Resonance and Sensory Information Processing: A Tutorial and Review of Application.” Clinical Neurophysiology 115, no. 2: 267–281. 10.1016/j.clinph.2003.09.014. 14744566

[ejsc70061-bib-0025] Mousavi, S. H. , A. Saberi Kakhki , D. Fazeli , L. Vogel , F. Horst , and W. I. Schöllhorn . 2024. “Effects of Contextual Interference and Differential Learning on Performance and Mental Representations in a Golf Putting Task.” European Journal of Sport Science 24, no. 3: 289–301. 10.1002/ejsc.12079.

[ejsc70061-bib-0026] Oftadeh, S. , A. Bahram , R. Yaali , F. Ghadiri , and W. I. Schöllhorn . 2021. “External Focus or Differential Learning: Is There an Additive Effect on Learning a Futsal Goal Kick?” International Journal of Environmental Research and Public Health 19, no. 1: 317. 10.3390/ijerph19010317. 35010577 PMC8751137

[ejsc70061-bib-0028] Porter, J. M. , and R. A. Magill . 2010. “Systematically Increasing Contextual Interference Is Beneficial for Learning Sport Skills.” Journal of Sports Sciences 28, no. 12: 1277–1285. 10.1080/02640414.2010.502946. 20845219

[ejsc70061-bib-0029] Ranganathan, R. , and K. M. Newell . 2010. “Motor Learning Through Induced Variability at the Task Goal and Execution Redundancy Levels.” Journal of Motor Behavior 42, no. 5: 307–316. 10.1080/00222895.2010.510542. 20826422

[ejsc70061-bib-0030] Ranganathan, R. , and K. M. Newell . 2013. “Changing up the Routine: Intervention‐Induced Variability in Motor Learning.” Exercise and Sport Sciences Reviews 41, no. 1: 64–70. 10.1097/jes.0b013e318259beb5. 23072823

[ejsc70061-bib-0032] Savelsbergh, G. J. , W. J. Kamper , J. Rabius , J. J. De Koning , and W. Schöllhorn . 2010. “A New Method to Learn to Start in Speed Skating: A Differencial Learning Approach.” International Journal of Sport Psychology 41, no. 4: 415. https://www.athleticskillsmodel.nl/wp‐content/uploads/2022/09/10_SavKaRaDeKoSch_anewmethodtolearntostartspeedskating_IntJSportPsychol_41_415‐4271.pdf.

[ejsc70061-bib-0033] Schmidt, M. , M. Kemena , and T. Jaitner . 2021. “Null Effects of Different Amounts of Task Variation in Both Contextual Interference and Differential Learning Paradigms.” Perceptual and Motor Skills 128, no. 4: 1836–1850. 10.1177/00315125211022302. 34078209

[ejsc70061-bib-0034] Schmidt, R. A. 1975. “A Schema Theory of Discrete Motor Skill Learning.” Psychological Review 82, no. 4: 225–260. 10.1037/h0076770.

[ejsc70061-bib-0035] Schmidt, R. A. , H. Zelaznik , B. Hawkins , J. S. Frank , and J. T. Quinn Jr . 1979. “Motor‐Output Variability: A Theory for the Accuracy of Rapid Motor Acts.” Psychological Review 86, no. 5: 415–451. 10.1037/0033-295x.86.5.415. 504536

[ejsc70061-bib-0036] Schöllhorn, W. 2005. “Differenzielles lehren und lernen von bewegung–durch veränderte annahmen zu neuen konsequenzen.” Zur Vernetzung von Forschung und Lehre in Biomechanik, Sportmotorik und Trainingswissenschaft 144, 125–135. https://www.bisp‐surf.de/Record/PU200606001478/availability#bdetails.

[ejsc70061-bib-0037] Schöllhorn, W. I. 2016. “Invited Commentary: Differential Learning Is Different From Contextual Interference Learning.” Human Movement Science 47: 240–245. 10.1016/j.humov.2015.11.018. 26872396

[ejsc70061-bib-0038] Schöllhorn, W. I. , H. Beckmann , M. Michelbrink , M. Sechelmann , M. Trockel , and K. Davids . 2006. “Does Noise Provide a Basis for the Unification of Motor Learning Theories?” International Journal of Sport Psychology 37, no. 2/3: 186. https://www.researchgate.net/publication/27466409_Does_noise_provide_a_basis_for_the_unification_of_motor_learning_theories.

[ejsc70061-bib-0039] Schöllhorn, W. I. , G. Mayer‐Kress , K. Newell , and M. Michelbrink . 2009. “Time Scales of Adaptive Behavior and Motor Learning in the Presence of Stochastic Perturbations.” Human Movement Science 28, no. 3: 319–333. 10.1016/j.humov.2008.10.005. 19062119

[ejsc70061-bib-0040] Schöllhorn, W. I. , M. Sechelmann , M. Trockel , and R. Westers . 2004. “Nie das Richtige trainieren, um richtig zu spielen.” Leistungssport 5, no. 2004: 13–17. https://sport.uni‐mainz.de/files/2014/05/tws_NiedasRichtigetrainieren.pdf.

[ejsc70061-bib-0041] Schöner, G. , and J. S. Kelso . 1988. “A Dynamic Pattern Theory of Behavioral Change.” Journal of Theoretical Biology 135, no. 4: 501–524. 10.1016/s0022-5193(88)80273-x.

[ejsc70061-bib-0042] Serrien, B. , B. Tassignon , J. Verschueren , R. Meeusen , and J.‐P. Baeyens . 2020. “Short‐Term Effects of Differential Learning and Contextual Interference in a Goalkeeper‐Like Task: Visuomotor Response Time and Motor Control.” European Journal of Sport Science 20, no. 8: 1061–1071. 10.1080/17461391.2019.1696894. 31755374

[ejsc70061-bib-0043] Shea, J. B. , and R. L. Morgan . 1979. “Contextual Interference Effects on the Acquisition, Retention, and Transfer of a Motor Skill.” Journal of Experimental Psychology: Human Learning & Memory 5, no. 2: 179–187. 10.1037/0278-7393.5.2.179.

[ejsc70061-bib-0045] Tassignon, B. , J. Verschueren , J.‐P. Baeyens , et al. 2021. “An Exploratory Meta‐Analytic Review on the Empirical Evidence of Differential Learning as an Enhanced Motor Learning Method.” Frontiers in Psychology 12: 533033. 10.3389/fpsyg.2021.533033. 34025487 PMC8138164

[ejsc70061-bib-0046] Wright, D. L. , and T. Kim . 2019. “Contextual Interference: New Findings, Insights, and Implications for Skill Acquisition.” In Skill Acquisition in Sport: Research, Theory and Practice (3rd ed.), edited by N. J. Hodges and A. M. Williams , 99–118. Routledge. 10.4324/9781351189750-6.

[ejsc70061-bib-0047] Wulf, G. , and R. Lewthwaite . 2016. “Optimizing Performance Through Intrinsic Motivation and Attention for Learning: The OPTIMAL Theory of Motor Learning.” Psychonomic Bulletin & Review 23, no. 5: 1382–1414. 10.3758/s13423-015-0999-9. 26833314

[ejsc70061-bib-0048] Wulf, G. , M. Raupach , and F. Pfeiffer . 2005. “Self‐Controlled Observational Practice Enhances Learning.” Research Quarterly for Exercise & Sport 76, no. 1: 107–111. 10.1080/02701367.2005.10599266. 15810775

